# Synthetic Attenuation Correction Maps for SPECT Imaging Using Deep Learning: A Study on Myocardial Perfusion Imaging

**DOI:** 10.3390/diagnostics13132214

**Published:** 2023-06-29

**Authors:** Mariana Andrea Prieto Canalejo, Aley Palau San Pedro, Ricardo Geronazzo, Daniel Mauricio Minsky, Luis Eduardo Juárez-Orozco, Mauro Namías

**Affiliations:** 1 Facultad Regional Buenos Aires, Universidad Tecnológica Nacional, Buenos Aires C1179AAS, Argentina; mprietocanalejo@frba.utn.edu.ar; 2 Fundación Centro Diagnóstico Nuclear (FCDN), Buenos Aires C1417CVE, Argentina; apalau@fcdn.org.ar; 3 Centro Atómico Constituyentes, Comisión Nacional de Energía Atómica, San Martín B1650LWP, Argentina; minsky@tandar.cnea.gov.ar; 4 Department of Cardiology, University Medical Center Utrecht, 3584 CX Utrecht, The Netherlands; l.e.juarez.orozco@gmail.com

**Keywords:** attenuation correction, deep learning, cardiac imaging, SPECT imaging

## Abstract

(1) Background: The CT-based attenuation correction of SPECT images is essential for obtaining accurate quantitative images in cardiovascular imaging. However, there are still many SPECT cameras without associated CT scanners throughout the world, especially in developing countries. Performing additional CT scans implies troublesome planning logistics and larger radiation doses for patients, making it a suboptimal solution. Deep learning (DL) offers a revolutionary way to generate complementary images for individual patients at a large scale. Hence, we aimed to generate linear attenuation coefficient maps from SPECT emission images reconstructed without attenuation correction using deep learning. (2) Methods: A total of 384 SPECT myocardial perfusion studies that used ^99m^Tc-sestamibi were included. A DL model based on a 2D U-Net architecture was trained using information from 312 patients. The quality of the generated synthetic attenuation correction maps (ACMs) and reconstructed emission values were evaluated using three metrics and compared to standard-of-care data using Bland–Altman plots. Finally, a quantitative evaluation of myocardial uptake was performed, followed by a semi-quantitative evaluation of myocardial perfusion. (3) Results: In a test set of 66 test patients, the ACM quality metrics were MSSIM = 0.97 ± 0.001 and NMAE = 3.08 ± 1.26 (%), and the reconstructed emission quality metrics were MSSIM = 0.99 ± 0.003 and NMAE = 0.23 ± 0.13 (%). The 95% limits of agreement (LoAs) at the voxel level for reconstructed SPECT images were: [−9.04; 9.00]%, and for the segment level, they were [−11; 10]%. The 95% LoAs for the Summed Stress Score values between the images reconstructed were [−2.8, 3.0]. When global perfusion scores were assessed, only 2 out of 66 patients showed changes in perfusion categories. (4) Conclusion: Deep learning can generate accurate attenuation correction maps from non-attenuation-corrected cardiac SPECT images. These high-quality attenuation maps are suitable for attenuation correction in myocardial perfusion SPECT imaging and could obviate the need for additional imaging in standalone SPECT scanners.

## 1. Introduction

Myocardial perfusion imaging (MPI), carried out via single-photon emission computed tomography (SPECT), is the most widely used non-invasive diagnostic technique for assessing blood flow in the myocardium. SPECT MPI offers high levels of sensitivity and specificity as well as good spatial resolution. The average sensitivity is 89% and the average specificity is 75% [1].

However, these images are vulnerable to attenuation artifacts (from factors including body size and shape, tissue composition and the presence of metal implants), which can lead to inaccurate interpretations and diagnoses in MPI studies [2,3]. The presence of attenuation artifacts caused by breast and diaphragmatic tissues can lead to the development of defects in the anterior and inferior walls, which are known to decrease the specificity of MPI [4,5]. There is also potential for a loss of sensitivity as perfusion defects may be interpreted as attenuation artifacts. A practical solution to minimize these artifacts is to scan the patient in a prone position, with improved specificity for evaluating inferior wall abnormalities by minimizing diaphragmatic attenuation [6]. However, performing CT-based attenuation correction leads to better sensitivity and specificity than prone-only imaging. The sensitivity is 100% and the specificity is 83.3% [7,8].

Traditionally, various methods have been implemented for the generation of attenuation correction maps (ACMs) in SPECT imaging, such as acquiring transmission data using external radiation sources or computed tomography (CT) scans [9]. These methods, however, are time-consuming and involve additional logistics and radiation exposure. The most commonly utilized method of acquiring CT-derived ACMs typically use bilinear transforms to convert Hounsfield units (HU) into linear attenuation coefficient maps [10]. One of the drawbacks of using CT for attenuation correction in SPECT/CT is the possibility of misalignment between emission and transmission scans due to the sequential acquisition of CT and SPECT and the possibility of patient motion. Thus, manual reregistration between the emission and transmission data is usually performed before reconstructing the SPECT images [11].

Due to the still relatively low availability of SPECT/CT equipment worldwide and the major interest in accelerating imaging protocols at the level of the individual, different methods (both analytic and iterative) have been proposed [12,13,14,15] to estimate the attenuation coefficients from emission data. One of the methods for attenuation correction in SPECT imaging is the use of iterative reconstruction algorithms that simultaneously estimate the activity and attenuation coefficients from the emission data. These methods are based on the maximum likelihood principle and can account for the scatter and attenuation effects in the emission data [16,17]. The use of iterative algorithms can also significantly improve the accuracy of attenuation correction, especially in challenging cases in which the attenuation maps are unavailable or inaccurate, by iteratively refining the estimates of both the activity and attenuation coefficients. However, they are limited by the introduction of artifacts, making them less precise when used to correct emission images. Nontrivially, they also require long computing times.

Recently, generative model derivation via deep learning has revolutionized image recognition and analysis [18]. It has been explored in medical imaging to obtain synthetic CT images [19,20] and corrected nuclear medicine images [21,22,23], suggesting its potential to improve image quality, reduce imaging time and minimize radiation exposure [24,25].

Several studies have shown the effectiveness of deep learning-based methods for attenuation correction in SPECT and PET (positron emission tomography) [26,27,28], including direct and indirect methods. Direct methods involve the use of a deep neural network to directly estimate the attenuation map from the emission data, while indirect methods require additional information, such as MRI or CT images, to estimate the attenuation map [29]. In a recent study, a generative adversarial network (GAN)-based method was proposed for attenuation correction in whole-body PET imaging [30]. The results showed that the GAN-based method outperformed traditional methods in terms of accuracy and reduced noise, indicating its potential for improving PET imaging. Similarly, a novel deep learning-based method was proposed for attenuation correction in SPECT imaging using convolutional neural networks [31]. The method used a convolutional neural network (CNN) to predict the attenuation correction map and was evaluated on SPECT data. The results showed that the deep learning-based method improved image quality and reduced the artifacts caused by attenuation, demonstrating its potential for clinical application.

Deep learning-based methods have shown promising results in quantitative SPECT imaging with attenuation correction [32,33,34]. In one study, a CNN based method was proposed for simultaneous attenuation and scatter correction in SPECT imaging [33]. Two studies proposed deep learning-based methods for attenuation correction in SPECT imaging using generative adversarial networks (GANs) [32,34]. The methods were evaluated on clinical cardiac SPECT data and showed improved image quality and quantitative accuracy. These studies demonstrate the potential of deep learning-based methods for attenuation correction in SPECT imaging, which can ultimately lead to more accurate and reliable diagnoses.

In the present study, we evaluated a deep learning approach for generating ACMs in MPI SPECT imaging, with an emphasis on the quantitative accuracy of the reconstructed SPECT images. These analyses build upon advances in the implementation of artificial intelligence in medical imaging to provide much-needed, efficient solutions for attenuation correction in MPI studies.

## 2. Materials and Methods

### 2.1. Data Acquisition and Preparation

In total, 384 myocardial perfusion studies that used ^99m^Tc-MIBI SPECT were retrospectively included. Normal and abnormal scans were recruited from both stress and rest tests performed at the FCDN (Fundación Centro Diagnostico Núclear). All patients provided their written informed consent, and the protocol was approved by the internal review board (Minutes No. 01/2022, 16 March 2022). The SPECT/CT images were obtained using a Millenium Hawkeye VG SPECT/CT system (GE Healthcare). This scanner has a dual-head SPECT system with low-energy and high-resolution (LEHR) collimators, plus a non-diagnostic single-slice CT scanner that rotates with the SPECT gantry. Of the patients, 65% were male. The average weight was 81 kg [40–170], the average body mass index was 29.1 kg/m^2^ [17.9–47.6] and the average age was 66 years [28–98]. The patients were injected with 444 MBq of ^99m^Tc-sestamibi for the first phase (usually stress) and 888 MBq for the second phase (usually rest) in a one-day protocol. Emission data were acquired with a 64 × 64 matrix, 30 angular steps over 90 degrees, with the detectors in L-mode, no zoom (voxel size = 6.9 mm) and 40 s per angular step. The photopeak energy window was 129.5–150.5 keV, and the lower scatter energy window was 111–119 keV. The CT was acquired with 140 kV at two revolutions per minute and a tube current of 2.5 mA.

The emission images were reconstructed using a 3D OSEM algorithm (ordered subsets expectation maximization) with three iterations, ten subsets, no scatter correction, no attenuation correction and resolution modeling relative to the detector distance (point-spread function (PSF)) with in-house reconstruction software [35] to generate non-attenuation corrected (NAC) images. The CT images were reconstructed using the scanner software. The CT images were converted from Hounsfield units (HUs) to ACMs at 140 keV, using a bilinear transform [10]. The ACMs were then co-registered to the NAC images using a translation-only transform (three degrees of freedom) with mutual information as a similarity metric, using the SimpleITK python library [36,37]. Co-registered ACMs were then used as targets for the U-Net output and to reconstruct attenuation and scatter corrected (AC-SC) images for validation purposes.

The reconstructed emission images (NAC or AC-SC) had a matrix size of 64 × 64 × 64, while the attenuation maps usually had a narrower axial field of view. Generally, the axial direction range possessed between 20 and 30 slices. Therefore, the SPECT images were trimmed in the z-axis direction for each patient, making the attenuation and emission information coincident.

The reconstructed SPECT image voxel intensity is proportional to the radiopharmaceutical tissue concentration. This intensity varies for each patient relative to the radiopharmaceutical doses, scanner sensitivity, the time of delay between the injection and acquisition and the patient’s weight, among other factors. We thus considered it appropriate to normalize the emission images to the range [0, 1] [38]. However, the ACMs were not normalized since they represent the tissue attenuation in cm^−1^ and are already included in the interval [0, 1] cm^−1^. The ACMs were filtered using a 13 mm FWHM Gaussian filter to better match the spatial resolutions of the reconstructed emission images and to compensate for residual misregistration defects.

### 2.2. Network Architecture and Training

**Figure 1:**
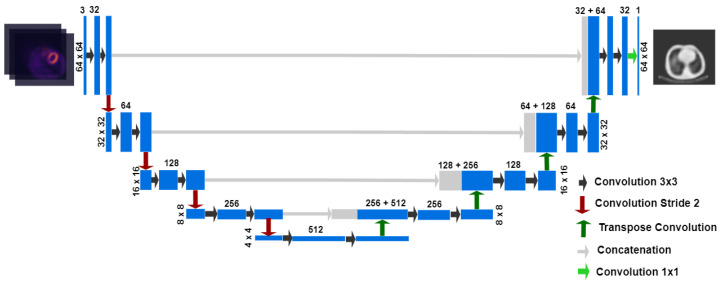
Convolutional deep neural network architecture, U-Net type. The input is an uncorrected 3-channel SPECT image, and the output is the ACM in cm^−1^.

The proposed deep learning (DL) approach consisted of a deep convolutional neural network (DCNN) based on a 2D U-Net architecture [39]. This architecture allows for the construction of generative models and has been used in the medical field to generate synthetic CTs [19,20]. This U-Net has five coder–decoder layers with symmetric concatenated connections (Figure 1).

Each layer uses 2D convolution blocks with 3 × 3 kernels, batch normalization (BN) [40], ReLu as activation function and dropout for regulation. Sub-sampling and oversampling steps were performed with stride 2 convolutions and transposed convolutions, respectively.

The loss function is presented in Equation (1). It combines the mean structural similarity index (MSSIM) metric with the mean relative error (MRE). The MSSIM (Equation (2)) allows for the assessment of the subjective visual appearance and is frequently used in image compression and restoration tasks [41]. On the other hand, the MRE (Equation (3)) measures the average percentage difference between the predicted and target values.

(1) $$L(Y,\hat{Y})=\alpha (1-MSSIM(Y,\hat{Y}))+(1-\alpha )MRE(Y,\hat{Y})$$

(2) $$MSSIM\left(Y,\hat{Y}\right)=\sum_{j=1}^{M}SSIM(y_{j},{\hat{y}}_{j})$$

(3) $$MRE(Y,\hat{Y})=\sum_{i=1}^{N}\left(\frac{\left|y_{i}-{\hat{y}}_{i}\right|}{y_{i}+\varepsilon }\right)$$

The SSIM metric evaluates the similarity of images by comparing local patterns and separating the evaluations of structure, luminance and contrast, as described in Equation (4).

(4) $$SSIM\left(x,y\right)={\left[l(x,y)\right]}^{\alpha }+{\left[c(x,y)\right]}^{\beta }+{\left[s(x,y)\right]}^{\gamma }$$

where the luminance, contrast and structure are defined as functions of the images’ means (*µ_x_* and *µ_y_*), their variance and covariance.

(5) $$l(x,y)=\frac{2\cdot \mu_{x}\cdot \mu_{y}+C_{1}}{{\mu_{x}}^{2}+{\mu_{y}}^{2}+C_{1}}$$

(6) $$c(x,y)=\frac{2\cdot \sigma_{x}\cdot \sigma_{y}+C_{2}}{{\sigma_{x}}^{2}+{\sigma_{y}}^{2}+C_{2}}$$

(7) $$s(x,y)=\frac{{2\cdot \sigma }_{xy}+C_{3}}{\sigma_{x}\cdot \sigma_{y}+C_{3}}$$

Given this independent comparison, the obtained results show good consistency with the qualitative visual appearances. Additionally, the luminance term implies a similarity between the mean values of the images.

The NAC images were ordered in the axial direction to provide the input to a convolutional neural network. The images adjacent to the target slice are added as additional channels in order to provide spatial information, obtaining a 64 × 64 × 3 input. Images from 254 patients were used as the training set, 64 images were used as the evaluation set, and the remaining 66 studies were used for external testing purposes.

To enhance the robustness and generalization performance of the network, data augmentation was applied. Data augmentation included random rotation along the z-axis (−5 ~ 5 degrees), random coronal, axial and sagittal flips, and random horizontal and vertical shifts (≤7 voxels). Finally, a random grid search was used for 1200 iterations to find the best hyperparameters for the model, which included the learning rate, the alpha parameter of the loss function, the optimizer function and the dropout rate.

### 2.3. Image Reconstruction for Validation

Emission data were reconstructed with an in-house 3D OSEM reconstruction algorithm with 3 iterations, 10 subsets, CT-derived ACMs, dual-energy window scatter correction and resolution modeling [35]. Two datasets were generated: one reconstructed with the measured linear attenuation coefficient maps and the other with the synthetic maps generated by the U-NET. The images were reconstructed in units proportional to photon counts (PtoPC).

### 2.4. Image Quality Metrics and Voxel-Level Quantitative Evaluation

The quality of the synthetic ACMs and the reconstructed emission data from 66 patients was evaluated using three metrics: MSSIM, MRE and the normalized mean absolute error (NMAE), which is defined as:

(8) $$NMAE\left(Y,\hat{Y}\right)=\frac{\sum_{i=1}^{N}\left|y_{i}-{\hat{y}}_{i}\right|}{N\left({Max}_{y}-{Min}_{y}\right)}$$

where *Max_y_* and *Min_y_* are the maximum and minimum values of the reference image, respectively, *N* is the total number of voxels, *y* is the reference image and $\hat{y}$ is the estimated image.

Differences in the linear attenuation coefficients from measured vs. synthetic ACMs and between reconstructed values in the AC-SC images were compared with Bland–Altman plots, and the 95% limits of agreement (LoAs) were estimated. The test patients were divided into male and female, with 61% being male and 39% female, and the quality of the reconstructed emission images was evaluated in each group separately.

### 2.5. Quantitative Evaluation of Myocardial Uptake

The reconstructed images from 66 patients were reoriented using Carimas 2.10 software (Turku PET Centre, Finland) to the three main cardiac axes by an experienced nuclear cardiologist. The myocardium region of interest (ROI) was automatically segmented using the built-in algorithm, after which a polar map with the uptake information for the 17 Standardized Myocardial Segments of the American Heart Association (AHA) was created [42]. The same reorientation and segmentation were applied to images reconstructed with the measured and the synthetic attenuation maps. Relative quantitative errors were estimated for each segment, for the global myocardial ROI and for the three vascular territories: the left anterior descending artery territory, divided into 1) without the apex (LDAwa) and 2) with the apex, the left circumflex artery (LCX) and the right coronary artery (RCA). The uptake values were compared using Bland–Altman plots.

### 2.6. Semi-Quantitative Evaluation of Myocardial Perfusion

The same 66 images from the test set were imported into a Xeleris 4.0 workstation (GE Healthcare) and analyzed using the Emory Cardiac Toolbox. Cardiac reorientation was performed manually by an experienced nuclear cardiologist, trying to use reorientation and processing parameters that were as similar as possible for images reconstructed with CT-based ACMs and U-Net-generated ACMs. However, in this software package, it is not possible to exactly replicate reorientation parameters between different datasets. An evaluation of left ventricular perfusion was used to calculate the perfusion score, considering both the extent and severity of ischemia in relation to the 17 segments of the polar map [43]. Normal perfusion is indicated on the scale as a score of 0 (normal perfusion in relation to the control group). Mild and moderate perfusion impairments are indicated by 1 and 2 points, respectively. A score of 3 points indicates significant perfusion impairment, while a score of 4 points is used to indicate total impairment, meaning practically no perfusion. The global scoring of myocardial perfusion uses the Summed Stress Score (SSS) and the Summed Rest Score (SRS). The SSS is the sum of the individual scores from the 17 segments of the polar map obtained during stress. When the SSS amounts to less than 4, the perfusion is considered normal or minimally abnormal (no significant perfusion disturbances); a result of 4–8 points indicates mildly abnormal perfusion, 9–13 points indicate moderately abnormal perfusion, and 13 or more points indicate the presence of significant extensive ischemia. For this study, we compared the SSS metrics obtained from both datasets with the Bland–Altman plots and estimated 95% LoAs. Using these categories, we also assessed changes in them when using synthetic ACMs. The percent myocardium abnormal (% Myo stress) was derived from normalized summed scores [44].

## 3. Results

### 3.1. Network Training and Sample Results

**Figure 2:**
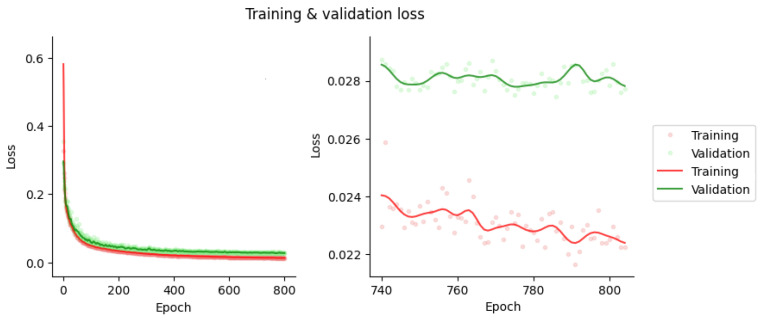
Training and validation losses for the U-Net Training.

The optimal hyperparameters were an Adam optimizer, a learning rate of 1 × 10^−4^, and value of 0.8, incorporating early stopping to prevent overfitting. Figure 2 illustrates the learning curves of the training and validation sets. The losses converged after 800 epochs of training to L = 2.28 × 10^−2^ and 2.82 × 10^−2^ for the training and validation sets, respectively.

**Figure 3:**
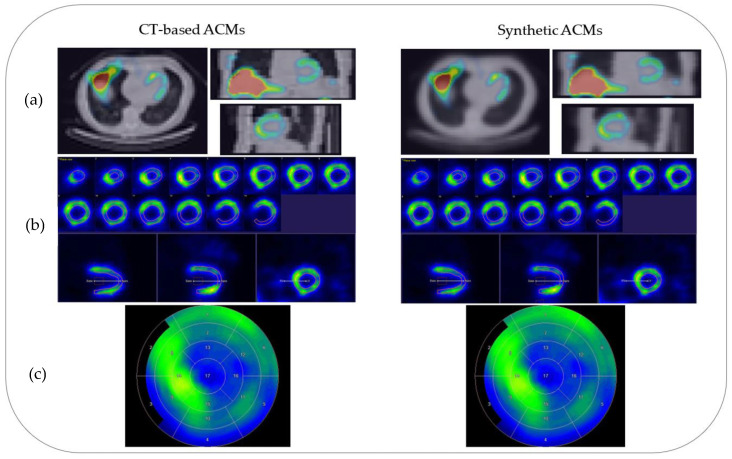
Sample results for a test patient. (**a**) AC-SC SPECT images fused with ACMs. (**b**) Short-axis slices. (**c**) Polar maps with 17 cardiac segments.

Figure 3 shows sample results of the generated ACMs and AC-SC images for a test patient.

### 3.2. Image Quality and Voxel-Level Evaluation

**Figure 4:**
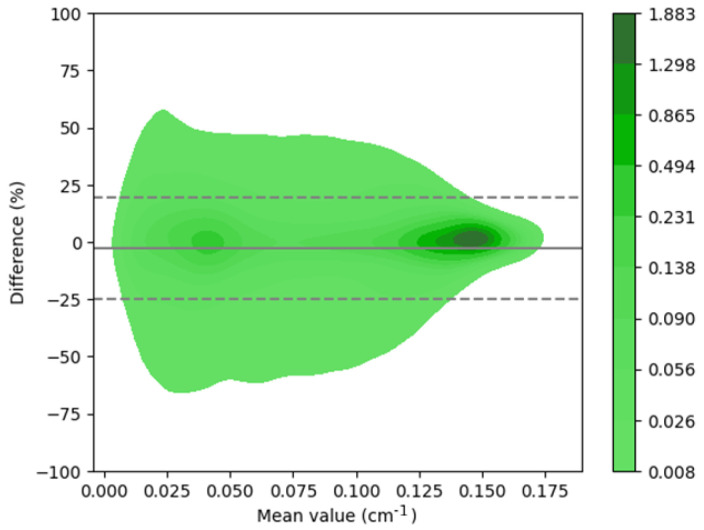
Bland–Altman plot for the synthetic vs. measured ACMs for the 66 test patients. Dotted lines represent the 95% LoAs, and the continuous line represents the bias value.

The image quality metrics for the synthetic ACMs of the 66 test patients are summarized in Table 1. Figure 4 shows the Bland–Altman plot for the errors of the synthetic ACM values vs. the CT-derived ACM values. The voxel-level 95% LoAs were [−23.49; 25.27]%, and the quantitative bias was −2.69%.

**Figure 5:**
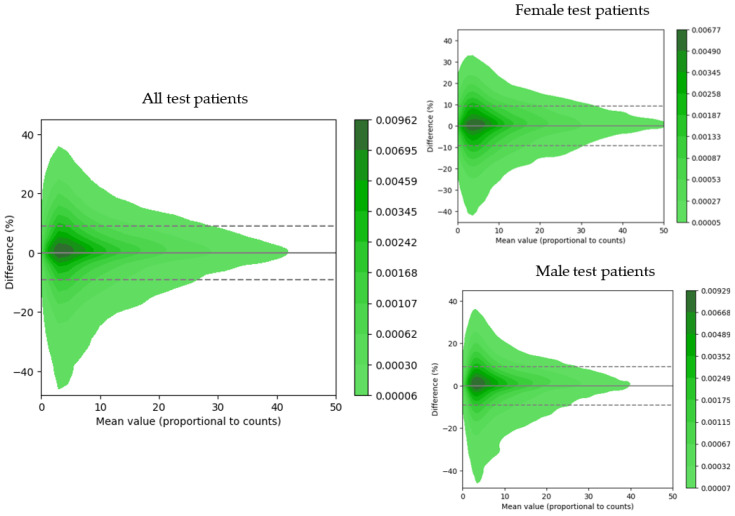
Bland–Altman plots of reconstructed emission values for the 66 test patients (synthetic ACMs vs. CT-based ACMs). Dotted lines represent the 95% LoAs, and the continuous line represents the bias value.

The evaluation of the image quality of the reconstructed AC-SC images of the 66 test patients with synthetic vs. CT-based ACMs was also performed using Bland–Altman plots (Figure 5). The 95% LoAs at the voxel level for the reconstructed SPECT images were [−9.04; 9.00]%, with a quantitative bias of −0.02% for all test patients; [−8.97; 8.91]%, with a quantitative bias of −0.03% for male patients; and [−9.27; 9.30]%, with a quantitative bias of −0.02% for female patients. The image quality metrics for the AC-SC images are summarized in Table 2.

### 3.3. Quantitative Evaluation of Myocardial Uptake

**Figure 6:**
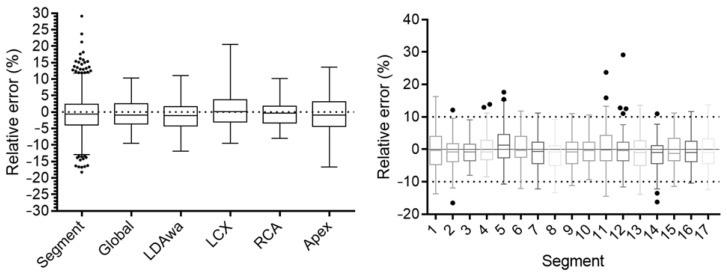
Boxplots of quantitative errors for reconstructed AC-SC SPECT images by vascular territory and segments in the 66 test patients. Whiskers show min–max values, the box shows the interquartile range, the circles represent outlier values and the line represents the median values.

Quantitative errors of the individual segment uptake, global uptake and the four vascular territories are shown in Figure 6 and summarized in Table 3. The 95% LoAs for segmental errors were [−11; 10]%. All territories had quantitative biases below 0.95%. The highest variance was found for the apical region (std = 5.8%), while the LDA, LCX and RCA standard deviation values were below 4.6%.

### 3.4. Semi-Quantitative Evaluation of Myocardial Perfusion

**Figure 7:**
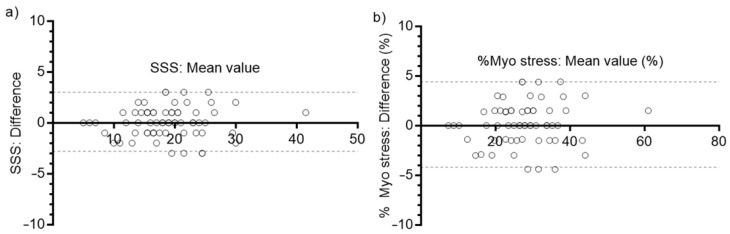
Bland–Altman plots for the 66 test patients: dotted lines represent the 95% LoAs and each circle represents a single data point (single patient) (**a**); SSS values (synthetic ACMs vs. CT-based ACMs). (**b**) Percent myocardium abnormal, derived from normalized summed scores.

The comparison of SSS values between images reconstructed with CT-based and synthetic ACMs is presented in Figure 7a. The bias was 0.076, and the 95% LoAs were [−2.8, 3.0]. Differences in percent myocardium abnormal are shown in Figure 7b. The bias was 0.11%, and the 95% LoA were [−4.2, 4.4]%.

When assesing global perfusion scores, 2 out of 66 patients (3.0%) showed changes in categories. One patient changed from significant extensive ischemia (SSS = 14) to moderately abnormal perfusion (SSS = 12), and the other changed from moderately abnormal perfusion (SSS = 9) to mildly abnormal perfusion (SSS = 8).

## 4. Discussion

Here, we implemented a generative DL model to synthesize ACMs from NAC SPECT images in MPI studies that used ^99m^Tc-sestamibi. These ACMs were then used for the attenuation correction of SPECT images.

Our approach provided improved quantitative results (Table 1 and Table 2) when compared to previously published topologies presented by Shi et al. (2020) [32] and Liu et al. (2022) [24]. In the first approach, various topologies were compared, obtaining the best metric as an NMAE of 0.26% ± 0.15%. In the second approach, with the post-reconstruction attenuation correction method, an NMAE of 1.1% ± 0.6% was achieved. However, our approach surpassed both, achieving an NMAE of 0.23% ± 0.13%. The utilized training strategy demonstrated consistent results with 254 training examples, which represents an order of magnitude smaller than the previously published results presented by Shanbhag et al. (2023) [34] where 4886 training examples were utilized. This can likely be explained by the tendency of the U-Net performance to saturate with increasing training sample sizes [45]. In Figure 2a, it can be observed that the proposed method was able to generate synthetic ACMs with high structural similarities (with an average MSSIM value of 0.97) when compared to the ground-truth CT-based ACMs despite having lower resolutions than the CT references. On the other hand, in Figure 3, it can be observed that the greater variability in synthetic attenuation coefficients compared to the real ones was found for low linear attenuation values. Since attenuation correction involves performing line integrals of attenuation coefficient maps, these differences for low linear attenuation values are not likely to significantly impact the correction process.

In the independent test data evaluation, the proposed U-Net yielded significantly low quantitative errors at both the voxel and image segment levels in the SPECT images corrected with synthetic ACMs (Table 2). The images in Figure 3b show how the short axis slices of the SPECT reconstructed images corrected using the CT-based attenuation map and the synthetic attenuation map are very similar, with a maximum error in LADwA of 0.7%. The reconstructed emission values had the highest relative errors for regions with low uptake (e.g., in the lungs) with respect to the myocardial regions with higher uptake (Figure 4). This suggests that areas with low uptake are more susceptible to attenuation correction artifacts when using synthetic ACMs. Notably, the performances in male and female patient images (Table 2 and Figure 4) were very similar and showed greater consistency than the study by Shi et al. (2020) [32], in which a bias of 2.6% was obtained for female subjects.

The segment-level quantitative errors (Table 3 and Figure 5) were of the same order as those obtained with a direct non-attenuation-corrected-to-attenuation-corrected image conversion approach (Yang et al., 2021, [22]), with the apex showing the highest quantitative errors. In addition, the 17-segment polar maps for each subject in Figure 2c (generated using Carimas 2.10 software) of the SPECT images corrected with both synthetic and CT-based ACMs were also consistent.

The semiquantitative evaluation of myocardial perfusion also resulted in good agreement between the CT-based vs. synthetic-ACM-based reconstructed images, with 95% LoAs for SSS values between [−2.8, 3.0] and a negligible bias (0.076). These differences probably receive a contribution from intra-operator variability since cardiac reorientation is a manual process, promoting the notion that residual errors are not due to the suboptimal performance of this novel approach. Only 2 patients out of 66 changed their global perfusion category, but the SSS values were already very close to the categorical boundaries. Finally, the 95% LoAs for percent abnormal myocardium were auspicious ([−4.2, 4.4]%).

Although we did not perform a comparative study, the use of a loss function that uses the MSSIM metric seems to be enough for good generative performance without the need of adversarial networks and losses, as proposed in previous works [23,32].

Our approach is the first reported method in Latin America, taking into consideration that the anatomical characteristics of this population may differ from others. Therefore, it is enriching to have a specific and tailored approach to these regional particularities in the clinical domain.

A limitation of our study is the relatively low quality of the CT scans used as references. These CT images can show artifacts from respiratory and cardiac motion because of the equipment’s low rotation speed (2 rpm) and beam-hardening artifacts from the presence of electrodes on the patient’s skin. However, this also suggests that our approach can be easily and possibly safely applied worldwide where needed and with similar results to those from real-world available images.

## 5. Conclusions

Generative deep learning can generate accurate attenuation correction maps from non-attenuation corrected cardiac SPECT images with ^99m^Tc-sestamibi. These high-quality attenuation maps are suitable for attenuation correction in MPI SPECT imaging and could obviate the need for additional imaging in standalone SPECT scanners.

## Figures and Tables

**Table 1:** ACM quality metrics for the 66 test patients.

	MSSIM	NMAE	MRE	MSE
Test	0.97 ± 0.01	3.08 ± 1.26 (%)	9.15 ± 3.7 (%)	1.2 × 10^−4^ ± 0.77 × 10^−4^ (cm^−1^)
Train	0.99 ± 0.01	1.04 ± 0.19 (%)	3.25 ± 0.98 (%)	0.1 × 10^−4^ ± 0.05 × 10^−4^ (cm^−1^)

**Table 2:** Reconstructed emission quality metrics for the 66 test patients.

	MSSIM	NMAE	MRE	MSE
All Test	0.99 ± 0.003	0.23 ± 0.13 (%)	5.9 ± 2.8 (%)	0.18 ± 0.26 (PtoPC)
Male Test	0.99 ± 0.002	0.26 ± 0.12 (%)	6.2 ± 2.7 (%)	0.16 ± 0.21 (PtoPC)
Female Test	0.99 ± 0.003	0.21 ± 0.12 (%)	5.9 ± 2.4 (%)	0.24 ± 0.34 (PtoPC)

**Table 3:** Quantitative errors by vascular territory (%) for the 66 test patients.

	Segment	Global	LDAwa	LCX	RCA	Apex
Number of values	1122	66	66	66	66	66
Minimum	−18	−9.5	−12.0	−9.5	−8.0	−17.0
25% Percentile	−3.9	−3.6	−4.2	−3.0	−3.3	−4.4
Median	−0.65	−1.0	−1.1	0.17	0.38	−0.95
75% Percentile	2.4	2.5	1.6	3.8	1.8	3.2
Maximum	29	10	11	9	10.0	14
Mean	−0.41	−0.61	−1.2	0.7	−0.37	−0.73
Std. Deviation	5.6	4.2	4.6	5.5	4.3	5.8

## Data Availability

Not applicable.
